# Pericarditis With Cardiac Tamponade Mimicking Yellow Nail Syndrome in a Patient With Rheumatoid Arthritis and a Paucity of Joint Symptoms

**DOI:** 10.7759/cureus.21523

**Published:** 2022-01-23

**Authors:** Ayuko Tokonami, Ryuichi Ohta, Yudai Tanaka, Shiho Amano, Chiaki Sano

**Affiliations:** 1 Family Medicine, Shimane University, Izumo, JPN; 2 Community Care, Unnan City Hospital, Unnan, JPN; 3 Community Medicine Management, Shimane University Faculty of Medicine, Izumo, JPN

**Keywords:** rural hospitals, pericardial effusion, edema, yellow nail syndrome, rheumatoid arthritis, rheumatoid pericarditis

## Abstract

Pericarditis is a cardiac disease that commonly manifests with rheumatoid arthritis, and its complications are related to rheumatoid arthritis disease activity. The diagnosis can be complicated in patients with multiple extra-joint complications of rheumatoid arthritis. We report a case of pericarditis in an 82-year-old woman with few joint symptoms who was admitted to the hospital due to worsening edema of the lower legs and dyspnea, which progressed to cardiac tamponade. The patient presented with gradual onset of edema of both lower limbs and bilateral pleural effusion and was initially diagnosed with yellow nail syndrome. Ultimately, the patient was diagnosed with rheumatoid pericarditis due to a rapid increase in pericardial effusion. She was treated with non-steroidal anti-inflammatory drugs (NSAIDs) and colchicine; however, the symptoms were progressive and required pericardiocentesis. After pericardiocentesis, the patient responded well to NSAIDs and colchicine, and systemic edema was relieved. This case highlights the fact that pericarditis associated with rheumatoid arthritis is not necessarily related to the severity of joint symptoms. Moreover, it can be difficult to differentiate pericarditis from multiple other diseases, such as yellow nail syndrome, in patients with rheumatoid arthritis who mainly have extra-articular symptoms.

## Introduction

Pericarditis is a common cardiac manifestation in patients with rheumatoid arthritis. Since the progression of this disease results in tamponade, which leads to a manifestation of symptoms, early detection of this condition is essential [1]. The first-line treatment of pericarditis involves the administration of colchicine and non-steroidal anti-inflammatory drugs (NSAIDs), and biologics are administered as second-line treatment; notably, treatment should be initiated early [2,3]. Rheumatoid arthritis-associated pericarditis has been linked to the disease activity of rheumatoid arthritis [4,5].

Various factors, including high levels of inflammatory proteins, such as C-reactive protein, and erythrocyte sedimentation rate, are associated with the onset of pericarditis among patients with rheumatoid arthritis [6]. In addition, elevated rheumatoid factor and anti-citrullinated peptide antibodies are considered risk factors for pericarditis [7]. To prevent the onset of pericarditis, it is necessary to control the disease activity of rheumatoid arthritis at an early stage. Therefore, the complication of pericarditis-associated rheumatoid arthritis could be related to the disease severity of rheumatoid arthritis, and individuals with more severe rheumatoid arthritis are at a greater risk of developing pericarditis [5,7]. However, in many cases, cardiac tamponade does not occur and the condition is relieved by continued drug treatment [8].

As there are extra-articular symptoms of rheumatoid arthritis other than pericarditis, it is often necessary to distinguish rheumatoid pericarditis from other diseases [5]. Various extra-articular symptoms combined with lower limb edema, nail discoloration, and deformity, for example, can sometimes lead to misdiagnosis as yellow nail syndrome [5,9].

In this report, we present the case of an 82-year-old woman who visited our hospital with the chief complaints of edema of both lower limbs, bilateral pleural effusion, and yellowing of the nails. Initially, the patient was diagnosed with yellow nail syndrome but was later diagnosed with pericarditis associated with rheumatoid arthritis. The symptoms progressed to cardiac tamponade due to low responsiveness to NSAIDs and colchicine. This case shows that rheumatoid arthritis disease activity alone does not predict the complication of pericarditis and highlights the need to distinguish the extra-articular symptoms of rheumatoid arthritis from other diseases such as yellow nail syndrome.

## Case presentation

**Figure 1:**
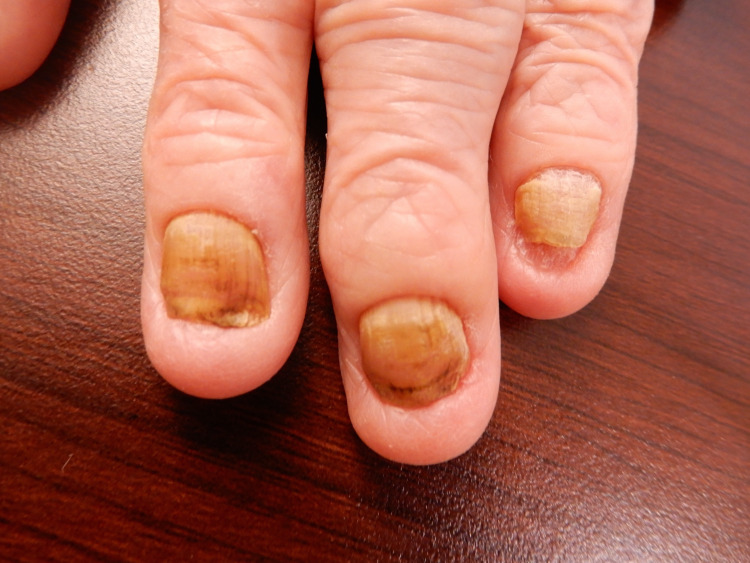
Yellow nails on the fingers of the patient

An independent 82-year-old woman presented to our hospital with chief complaints of bilateral leg edema three months prior to the admission. She had severe proteinemia, chronic bronchitis, bilateral pleural effusion, and yellow nails (Figure 1).

The patient was diagnosed with yellow nail syndrome and was followed up at the outpatient clinic with frusemide at 20 mg/day. Her symptoms worsened with exertional dyspnea and systemic edema, and she was hospitalized for investigation. The patient’s medical history indicated that she had been diagnosed with rheumatoid arthritis seven years prior and treated with prednisolone 5 mg, bucillamine 200 mg, salazosulfapyridine 1,000 mg, and iglamotide 50 mg. Her medical history also included diabetes, dyslipidemia, chronic sinusitis, chronic bronchitis, and hypothyroidism without heart failure.

**Figure 2:**
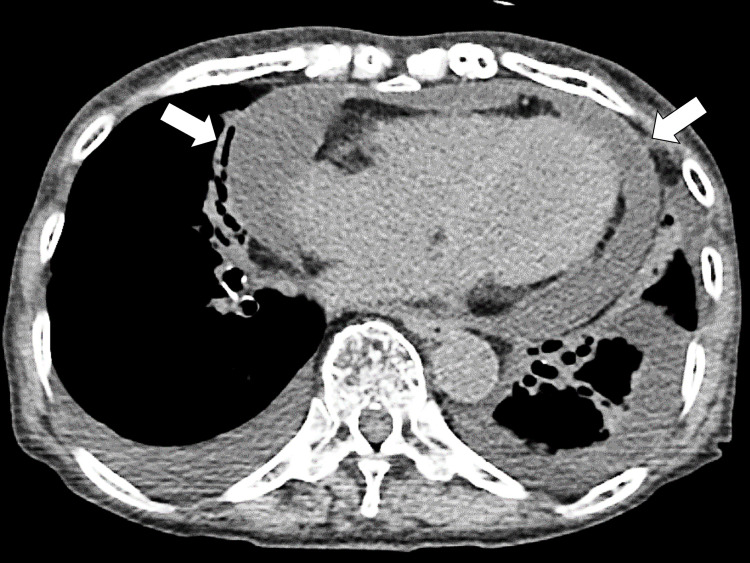
Pericardial effusion on the initial CT (arrows) CT: computed tomography

Upon admission, her vital signs were as follows: blood pressure of 124/60 mmHg; pulse rate of 72/minute, respiratory rate of 16/minute, body temperature of 37.4 °C, and oxygen saturation of 97% on room air. Physical examination revealed bilateral expiratory wheezing and pitting edema in the arms and legs but no joint tenderness with effusions. Chest CT on admission showed bilateral pleural and pericardial effusion (Figure 2).

CT did not show any lymphadenopathy (>1 cm) or masses. Blood tests at admission showed no signs of thyroid abnormalities (Table 1).

**Table 1:** Initial laboratory data of the patient HCV: hepatitis C virus; HIV: human immunodeficiency virus; HBs: hepatitis B surface antigen; HBc: hepatitis B core antigen; SARS-CoV-2: severe acute respiratory syndrome coronavirus 2

Marker	Level	Reference
White blood cells	5.9	3.5–9.1 × 10^3^/μL
Neutrophils	78.4	44.0–72.0%
Lymphocytes	16.3	18.0–59.0%
Monocytes	7.9	0.0–12.0%
Eosinophils	0.6	0.0–10.0%
Basophils	0.4	0.0–3.0%
Red blood cells	3.57	3.76–5.50 × 10^6^/μL
Hemoglobin	10.1	11.3–15.2 g/dL
Hematocrit	31.7	33.4–44.9%
Mean corpuscular volume	88.8	79.0–100.0 fl
Platelets	17.9	13.0–36.9 × 10^4^/μL
Prothrombin time-international normalized ratio	1.4	0.90~1.15
Activated partial thromboplastin time	35	25–40 s
Fibrinogen	235	200–400 mg/dL
Erythrocyte sedimentation rate	82	2–10 mm/h
Total protein	6.2	6.5–8.3 g/dL
Albumin	2.7	3.8–5.3 g/dL
Total bilirubin	0.3	0.2–1.2 mg/dL
Direct bilirubin	0.1	0–0.4 mg/dL
Aspartate aminotransferase	47	8–38 IU/L
Alanine aminotransferase	36	4–43 IU/L
Alkaline phosphatase	196	106–322 U/L
γ-Glutamyl transpeptidase	76	<48 IU/L
Lactate dehydrogenase	315	121–245 U/L
Uric acid	6.2	3.0–6.9 mg/dL
Blood urea nitrogen	36.6	8–20 mg/dL
Creatinine	1.12	0.40–1.10 mg/dL
Estimated glomerular filtration rate	35.7	>60.0 mL/min/L
Serum sodium	141	135–150 mEq/L
Serum potassium	3.8	3.5–5.3 mEq/L
Serum chloride	106	98–110 mEq/L
Serum calcium	8.3	3.5–5.3 mg/dL
Serum phosphorus	3.3	0.2–1.2 mg/dL
Ferritin	142.1	14.4–303.7 ng/mL
Creatinine kinase	91	56–244 U/L
C-reactive protein	7.78	<0.30 mg/dL
Thyroid-stimulating hormone	10.3	0.35–4.94 μIU/mL
Free T4	1.4	0.70–1.48 ng/dL
Vitamin B1	28	21.3–81.9 pg/mL
Folic acid	8.2	>4.0 ng/mL
Immunoglobin G	1,004	870–1,700 mg/dL
Immunoglobin M	87	35–220 mg/dL
Immunoglobin A	408	110–410 mg/dL
Immunoglobin E	171	<173 mg/dL
HBs antigen	0	IU/mL
HBs antibody	0	mIU/mL
HBc antibody	0	S/CO
HCV antibody	0	S/CO
Syphilis treponema antibody	0	S/CO
SARS-CoV-2 antigen	Negative	
Antinuclear antibody	<40	<40
Homogeneous	(-)	
Speckled	(-)	
Nucleolar	(-)	
Peripheral	(-)	
Discrete	(-)	
Cytoplasm	(-)	
Proteinase 3-anti-neutrophil cytoplasmic antibody	<1.0	<1.0 U/mL
Myeloperoxidase-anti-neutrophil cytoplasmic antibody	<1.0	<1.0 U/mL
Anti-SS-A antibody	<1.0	<1.0 U/mL
Anti-SS-B antibody	<1.0	<1.0 U/mL
Anti-ds-DNA IgG antibody	<10	IU/mL
Rheumatoid factor	144	<15 U/mL
Anti-citrullinated peptide antibody	>500	<5 U/mL
Beta-D-glucan	11	<20 pg/mL
interferon-gamma release assays	(-)	

**Figure 3:**
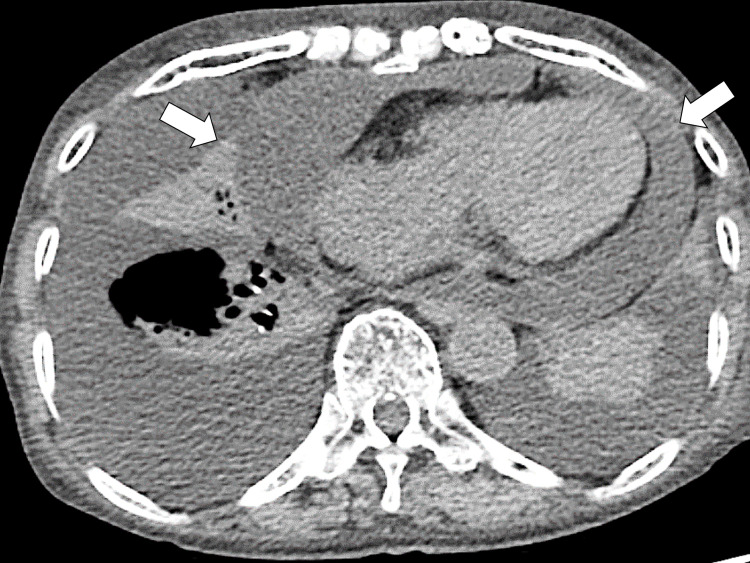
Cardiac tamponade on the subsequent CT (arrows) CT: computed tomography

Based on the clinical presentation and imaging and laboratory data, the patient was diagnosed with acute pericarditis and treated with colchicine (1.2 mg/day) and diclofenac (75 mg/day). On the sixth day of admission, systemic edema and dyspnea worsened, and a decrease in urine volume was observed even with the intravenous use of furosemide (60 mg/day). A follow-up CT showed an increase in pericardial effusion and compression of the heart (Figure 3).

**Figure 4:**
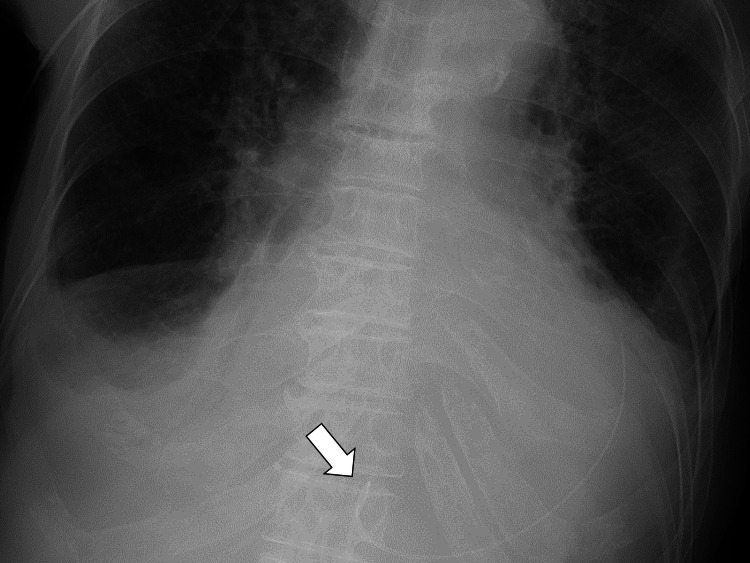
Pericardial drainage catheter for pericardiocentesis (arrow)

Based on the clinical presentation with lowering blood pressure, she was diagnosed with cardiac tamponade. Pericardiocentesis and pericardial drainage were performed given the possibility of cardiac tamponade (Figure 4).

The pericardial fluid was dark red, with a fluid concentration of protein of 3.9 g/dL, lactate dehydrogenase of 487 IU/L, albumin of 1.8 g/dL, adenosine deaminase of 13.5 U/L, and glucose of 223 mg/dL. Cytology and culture of the pericardial effusion were negative for malignancy and infection, respectively. The etiology was considered exudative and accompanied by inflammation. Based on the clinical presentation and imaging and laboratory data, the patient was diagnosed with rheumatoid pericarditis after excluding malignant tumors given the absence of malignant findings upon examining pericardial effusions and tuberculosis based on the low ADA level. In addition, we excluded fungal infections and wet beriberi based on negative culture test results and a normal vitamin B1 level, respectively.

**Figure 5:**
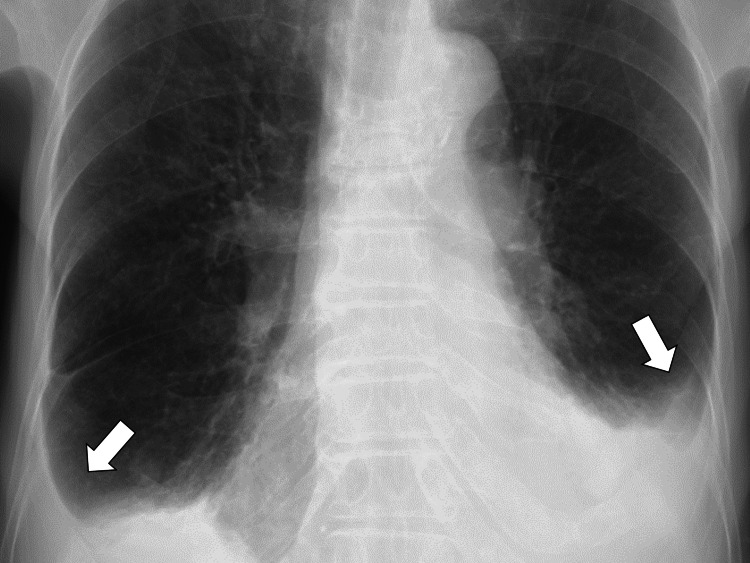
Pericardial drainage catheter for pericardiocentesis (arrows)

Respiratory distress improved after a pericardial drain was placed; peripheral perfusion also improved, and urine output increased. Two days after the placement of the pericardial drain, there was almost no residual pericardial fluid with an ejection fraction of 0.6, which was confirmed by transthoracic echocardiography. The drainage volume was 2.5 mL/day, and the drain was removed. Pleural effusion and lung infiltration on chest radiography increased after pericardiocentesis, suggesting temporary exacerbation of left heart failure with preserved ejection fraction (Figure 5).

The patient was treated with intravenous furosemide (40 mg/day), which alleviated her symptoms. No recurrence of pericarditis was observed during the subsequent course.

On day 10 after admission, swelling and tenderness were observed on the left wrist and right knee joints. The synovial fluid obtained by arthrocentesis of the right knee joint was serosanguinous; however, sodium urate and calcium pyrophosphate were not detected. Joint symptoms were attributed to rheumatoid arthritis. To control the symptoms, a subcutaneous injection of tocilizumab 162 mg was initiated. In the following days, her symptoms improved, and she was discharged home in an independent condition.

## Discussion

We reported a case of cardiac tamponade from pericarditis associated with rheumatoid arthritis, with no significant joint symptoms. It was difficult to distinguish yellow nail syndrome from rheumatoid arthritis due to similar extra-articular presentations in our patient. Pericarditis associated with rheumatoid arthritis is not necessarily related to the severity of joint symptoms. In patients with rheumatoid arthritis who have extra-articular symptoms predominantly, it has been suggested that it is difficult to distinguish rheumatoid pericarditis from various other diseases [7,9].

This case of pericarditis in a patient with rheumatoid arthritis who had clinically mild joint symptoms suggests the importance of monitoring the daily symptoms in these patients. In general, complications of rheumatoid arthritis-associated pericarditis are related to the disease activity of rheumatoid arthritis [7]. When a patient with poorly controlled rheumatoid arthritis complains of symptoms, such as chest pain, dyspnea, and lower leg edema, it is necessary to proceed with a systematic and detailed evaluation to rule out pericarditis [10]. In this case, it may have been difficult to suspect the complication of pericarditis because the joint symptoms were not critical.

Although swelling of both wrist joints and an increase in inflammatory response were observed during the treatment course of pericarditis, these symptoms were not detected during the outpatient follow-up. With the onset of pericarditis, rheumatoid arthritis control may have become poor in our patient. Previous studies suggest that pericarditis complication is a factor that indicates poor control of rheumatoid arthritis [11,12]. In this case, the onset of pericarditis could have been a precursor to the exacerbation of rheumatoid arthritis. Moreover, the triggers of pericarditis in patients with rheumatoid arthritis have not been clarified [12,13]. In previous studies, in addition to the disease activity of rheumatoid arthritis, aging was also associated with pericarditis [14,15]. Since this patient was also elderly, age could have been a trigger for the onset. In the future, it will be necessary to investigate the triggers of pericarditis in patients with rheumatoid arthritis.

Furthermore, multiple extra-articular symptoms of rheumatoid arthritis can render the diagnosis of pericarditis challenging; therefore, it is necessary to carefully distinguish between various diseases with a similar presentation, such as yellow nail syndrome and pericarditis [5,16]. In our case, the patient had chronic sinusitis and bronchitis related to rheumatoid arthritis. The exacerbation of the patient’s lower leg edema and pleural effusion was initially diagnosed as yellow nail syndrome, and the patient was discharged for follow-up, despite these being the initial symptoms of pericarditis. It cannot be denied that the patient had yellow nail syndrome based on the presence of yellow nails, possible lymphedema, chronic sinusitis, and bilateral pleural effusion. In contrast, acute exacerbation of lower leg edema was observed over a one-month span; therefore, the course differed from the progression of yellow nail syndrome.

For the effective diagnosis of rheumatoid pericarditis, close follow-up and a high index of suspicion are essential in patients with multiple complications of rheumatoid arthritis. Additionally, a methodical and stepwise approach is required to rule out the differentials [16]. Many non-fatal complications of rheumatoid arthritis can progress chronically [5]. In contrast, pericarditis may acutely manifest and progress to fatal conditions, such as cardiac tamponade, as in this case [3,11]. Older people in rural settings tend to manage their symptoms by themselves [17-20]. If a patient with rheumatoid arthritis with extra-articular symptoms experiences lower leg edema, paying careful attention to the rate of progression of symptoms for early diagnosis and treatment of pericarditis can prevent progression to a fatal condition. Hence, older patients with rheumatoid arthritis should be informed as to when they should visit physicians quickly.

## Conclusions

Lower leg edema in patients with chronic rheumatoid arthritis and multiple associated extra-articular symptoms require thorough differential diagnoses. Differential diagnoses, such as yellow nail syndrome, may be based on multiple extra-articular symptoms. Therefore, it is important to consider the differential diagnoses according to the rate of progression of symptoms. In particular, if acute progressive lower leg edema is observed, it is important to consider the complication of pericarditis and initiate early diagnosis and drug treatment.
